# Nurse prescribing right: a bibliometric analysis

**DOI:** 10.3389/fmed.2025.1417376

**Published:** 2025-08-08

**Authors:** Yingying Wang, Ruixiao Jia, Su Gu, Zihan Wang, Liqun Zhu, Shaoyong Ma

**Affiliations:** ^1^Nursing Department, Affiliated Hospital of Jiangsu University, Zhenjiang, Jiangsu, China; ^2^Department of Emergency, Yijishan Hospital of Wannan Medical College, Wuhu, Anhui, China; ^3^Gastrointestinal Surgery, Zhejiang Cancer Hospital, Hangzhou, Zhejiang, China; ^4^The First People’s Hospital of Yancheng, Yancheng, Jiangsu, China; ^5^Nursing Department, The First Affiliated Hospital of Anhui Medical University, Hefei, Anhui, China; ^6^School of Nursing, Wannan Medical College, Wuhu, Anhui, China

**Keywords:** nurse prescription, VOSviewer, CiteSpace, bibliometric analysis, visual analysis

## Abstract

**Background:**

The concept of nurse prescribing rights is undergoing rapid evolution, and there is increasing interest in academic circles regarding research on this topic.

**Objective:**

This study aims to conduct a comprehensive bibliometric analysis of nurses’ prescribing rights to understand the research background, emerging trends, and relevant academic impacts in this field, so as to provide valuable insights for the formulation of health policies and the promotion of more equitable and efficient healthcare services.

**Methods:**

We conducted a bibliometric analysis of the articles on nurses’ prescribing rights from the establishment of the database until 6 December 2023. The data used were collected from the Web of Science Core Collection database. We carried out a bibliometric analysis with the aim of determining the overall research directions and trends of the currently published literature. At the same time, we sought to identify the most productive and influential countries/regions, institutions, journals, authors, research categories, keywords, and articles in the field of nurses’ prescribing rights. We used software such as VOSviewer and CiteSpace, as well as the tool Biblioshiny to collect and analyze the data, with a focus on conducting analysis and visualization.

**Results:**

A total of 555 papers were obtained from 848 institutions in 44 countries. These papers were published in 237 journals and were written by 1,902 authors. From 2018 to 2023, there was a significant increase in the number of publications, accounting for 40.54% (225/555) of the total. The United States and the University of Reading have published the largest number of papers on this topic. The United Kingdom has the highest frequency of citation and cooperation. Out of the 555 publications, six clusters were formed, focusing on Nursing Practitioners, Primary Care, and Education. The research frontier mainly revolves around Buprenorphine and Opioid.

**Conclusion:**

This study provides a comprehensive overview of the research progress in the field of nurse prescribing rights. Through statistical analysis and network visualization, we have identified the background, trends, and key topics in this research area. Our findings indicate that the prescribing rights of nurses have experienced rapid development in recent years and have played a crucial role in addressing the shortage of primary medical resources. The insights gained from this study can serve as a valuable reference and provide guidance for future research in this field.

## Introduction

Prescription rights refer to the legal authority granted to healthcare professionals to prescribe medications, and these rights may vary depending on the laws governing their practice (1). In order to meet the increasing demands of healthcare services due to aging populations and the rise in long-term conditions, health systems often implement measures such as role extension, expansion of health professional roles, and task redistribution and empowerment (2). These measures aim to address the challenges faced by contemporary health systems. The shortage of medical services in the United States in the 1960s prompted the consideration of expanding the role of nurses, including granting them the right to prescribe. Regulated nurse prescribing was introduced in the United States in the 1970s and has since been extended to all regions of the World Health Organization (WHO) (3). In 2001, during the fifth meeting of the Advanced Practice Nursing and Nurse Prescription: Impact on Regulation, Nursing Education, and Practice in the Eastern Mediterranean, the WHO urged countries worldwide to empower nurses with appropriate prescribing rights to enhance the provision of healthcare services (4). In low- and middle-income countries, granting nurses prescribing rights can provide patients with more convenient and timely medical services, particularly in areas with relatively scarce medical resources (4). However, implementing nurses’ prescribing rights in these countries also faces several challenges, such as uneven nursing education levels, imperfect regulatory mechanisms, and constraints from social norms. These issues need to be addressed step by step through measures such as strengthening education and improving relevant laws and regulatory systems to ensure the safe and effective implementation of nurses’ prescribing rights. The policy priorities of WHO’s Global Strategic Directions for Care and Midwifery 2021–2025 are focused on advancing nurse prescribing (5). This includes delegating appropriate prescribing authority to nurses, which not only enhances access to high-quality nursing services but also promotes convenience. Nurse prescribing is of significant practical importance given the current healthcare cost control and task replacement landscape (6). The field of nurse prescribing continues to advance, with an increasing number of relevant studies being conducted in recent years. Comprehensive literature searches and summaries are crucial in this regard. Bibliometric methods, which combine quantitative and qualitative approaches, are employed to describe, evaluate, and predict the current status and future trends of scientific and technological research in specific fields (7). Bibliometric analysis is a method that utilizes various software systems to minimize bias in manual analysis (8). However, there is currently a lack of bibliometric analysis or follow-up studies on nurse prescriptions. Therefore, our objective is to conduct a comprehensive analysis of the research status of nurses’ prescribing rights and identify the latest trends in this area using papers published in the Web of Science (WOS) database. We aim to provide a basis for policy promotion in regions where prescribing rights have not yet been implemented and to offer references for the implementation and development of nurses’ prescribing rights in the context of whole life cycle health management services.

## Materials and methods

### Data sources and search strategy

**FIGURE 1:**
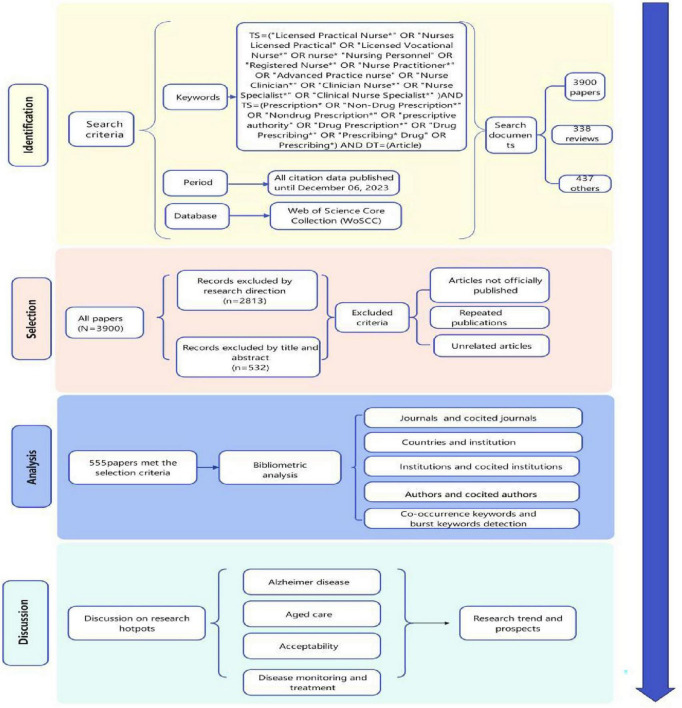
Flow chart of the literature screening process and research framework.

On 6 December 2023, all citation data published until 6 December 2023, were retrieved from the Web of Science Core Collection (WoSCC). The data were independently verified by two authors (Gusu and Wongyingying). The detailed search string is listed in Figure 1 and outlined as follows.

First, we retrieved articles with the keyword “nurse” and derivative keywords of the topic. The query command for #1 was TS = (“nurse*” OR “Nursing Personnel” OR “Registered Nurse*” or “Licensed Practical Nurse*” OR “Nurses Licensed Practical” OR “Licensed Vocational Nurse*” OR “Nurse Practitioner*” OR “Advanced Practice nurse*” OR “Nurse Clinician*” OR “Clinician Nurse*” OR “Nurse Specialist*” OR “Clinical Nurse Specialist*”). TS refers to the topic tag for search string retrievals.

Second, we retrieved articles with the keyword “Prescription.” The query command for #2 was TS = (“Prescription*” OR “Non-Drug Prescription*” OR “Non-drug Prescription*” OR “prescriptive authority” OR “Drug Prescription*” OR “Drug Prescribing*” OR “Prescribing*”).

Third, we combined the commands above (#1 AND #2) AND Article. This was the last step of the screening process. The query command (#1 AND #2) indicates retrieving papers related to both nurse and prescription. This refers to articles that study literacy in the field of nurse prescribing.

Fourth, we retrieved articles with the document types “article.” The query command for #4 was (#3 AND DT = (Article)). The document type was article. The language was not limited.

Inclusion criteria were: (a) Published articles on nurses prescribing or reviews on nurses prescribing; (b) articles published until 6 December 2023; and (c) articles retrieved from the WoSCC.

Exclusion criteria were: (a) articles not officially published; (b) conference abstracts and proceedings, corrigendum documents; (c) repeated publications; and (d) unrelated articles.

Finally, a total of 555 literatures were retrieved, and all were exported in “full record and reference format” and saved in “plain text format.” The parameters of CiteSpace were as follows: time slicing (until 6 December 2023), years per slice (1), term source (all selection), node type (choose one at a time), top *n* = 50 and top *n*% = 50. From each publication, we gathered the following basic data: title, abstract, authors, institution, country or region, journal, keywords, and references.

The literature retrieved will be initially screened by the research group according to the inclusion and exclusion criteria based on the titles and abstracts. If necessary, the full texts will be read for screening. The retrieval results will be exported in plain text format, and the exported fields will be “full record and cited references.” After exporting the literature in Refworks format, it will be saved as a TXT file. The exported file will be format-converted and analyzed in the Citespace 6.2.R2 software. The retrieved data were collected on 6 December 2023, to minimize bias caused by daily updates. The search results were exported as “complete records and references,” including titles, authors, institutions, abstracts, journals, publication dates, and other information.

In this study, we utilized several tools to analyze the literature.

VOSviewer 1.6.18, a free Java document mapping software developed by the Science and Technology Research Center of Leiden University in the Netherlands, was employed for analyzing collaborative networks and keyword co-occurrence networks. The collaborative network between countries/regions was presented using the Bibliometrix online analysis platform (9, 10). Furthermore, we analyzed the network map of scientific cooperation between countries/regions using the same platform. To identify burst keywords, we utilized Citespace 6.2.R2, a scientometric tool developed by Chen (11).

## Results

### General trends in publications and citations

**FIGURE 2:**
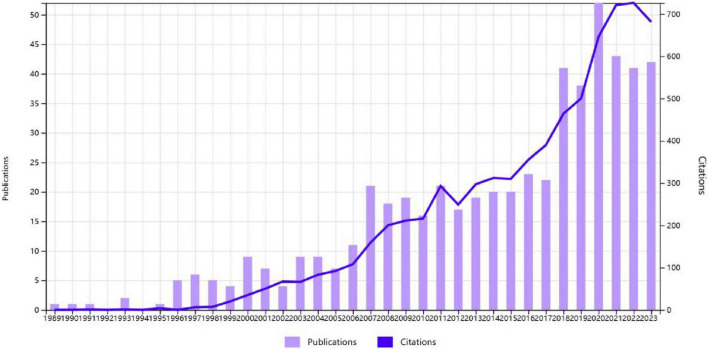
Total publications and citations from 1989 to 2023.

Figure 2 illustrates the trends in publications and citations concerning nurse prescribing rights. Our search yielded a total of 555 articles published from 1989 to 2023. The number of published articles has consistently increased over time, indicating an evident overall growth trend. Based on the annual average publication calculation, the development trend can be divided into three stages: 1. Initial stage (1989–2006): During this period, the number of published articles remained in single digits, with an average of around five articles per year. 2. Slow growth stage (2007–2017): From 2007 to 2017, the field experienced a gradual growth stage, with the annual number of publications consistently below 25. The average number of papers published per year was approximately 20. 3. Rapid growth stage (2018–2023): Since 2018, there has been a significant increase in the study of nurse prescribing rights, with an average of 45 articles published per year. The highest peak occurred in 2020, with 52 articles (9.37% of the total). A similar trend can be observed in citations, indicating the growing importance and potential impact of the field. The year 2007 marked a turning point from the initial stage to the slow growth stage, while 2018 was a key year for transitioning from the slow growth stage to rapid development. These findings suggest the presence of significant literature in 2007 and 2018, which aligns with our study’s results. In a study conducted by Bradley and Nolan (12), interviews were conducted with 45 prescribing nurses between 2005 and 2006. The findings indicated that nurse prescribing enhanced nurses’ knowledge of medicines, job satisfaction, patient service care, and confidence in participating in prescribing decisions within the medical team. Similarly, Traczynski and Udalova (13) found that independent prescribing by nurses increased the frequency of routine outpatient visits, improved the quality of care, and reduced the utilization of emergency rooms. It is worth noting that policy support can greatly facilitate the rapid development of a specific field. For example, in 2017, the United States implemented the Comprehensive Addiction and Recovery Act, which permits nurse practitioners to prescribe buprenorphine (14). This policy has played a significant role in promoting the swift advancement of research on nurses’ prescribing rights.

### Country/regional and institutional analysis

A total of 44 countries/regions and 848 institutions participated in the publication of 555 papers. The publication rankings of the top 10 countries/regions are presented in Table 1. The United States had the highest number of publications during the study period (*n* = 234, 42.16%), followed by England (*n* = 132, 23.78%) and Australia (*n* = 46, 8.29%). Among the top 10 countries/regions, more than 95.00% of all papers were published, indicating uneven research development across countries/regions. Despite the United States surpassing the United Kingdom in terms of the number of articles published, it falls behind in literature citations and the intensity of cooperative links compared to the United Kingdom. Revised: A total of 44 countries/regions and 848 institutions participated in the publication of 555 papers. Table 1 presents the publication rankings of the top 10 countries/regions. The United States had the highest number of publications during the study period (*n* = 234, 42.16%), followed by England (*n* = 132, 23.78%) and Australia (*n* = 46, 8.29%). Amongst these top 10 countries/regions, over 95.00% of all papers were published, indicating varying levels of research development across different regions. Despite the United States leading in terms of the number of articles published, it lags behind the United Kingdom in terms of literature citations and the intensity of cooperative links.

**TABLE 1:** Top 10 countries/regions by number of publications.

Rank	Countries/regions	Publications, *n* (%)	Citations	Total link strength
1	United States	234 (42.16)	2,395	16
2	England	132 (23.78)	2,877	60
3	Australia	46 (8.29)	565	21
4	Canada	23 (4.14)	174	20
5	Scotland	22 (3.96)	280	10
6	Spain	19 (3.43)	75	22
7	Netherlands	17 (3.06)	257	21
8	New Zealand	17 (3.06)	451	5
9	Wales	17 (3.06)	399	29
10	Poland	15 (2.70)	105	27

**FIGURE 3:**
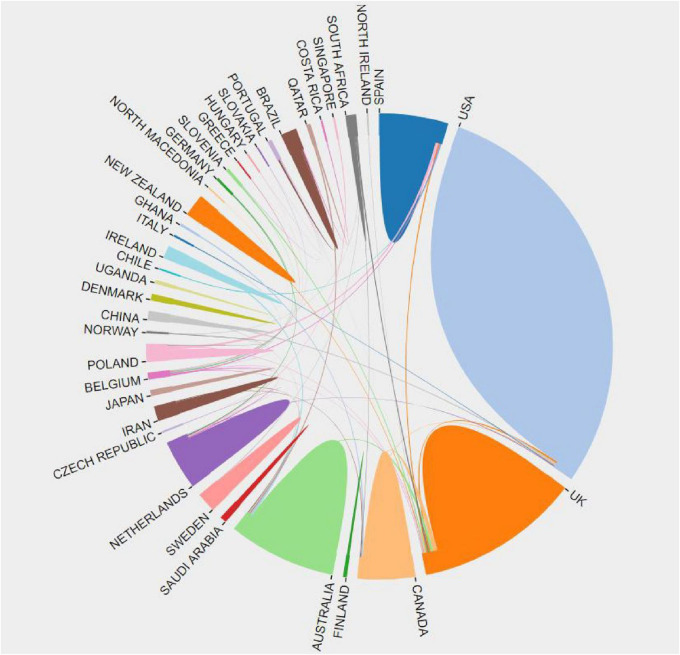
Map of national/regional scientific cooperation networks.

The research paper mentioned a total of 44 countries or regions (Figure 3). These countries or regions were depicted using blocks of various colors, where the size of each block represented the number of publications. Moreover, the size of the covered areas indicated the strength of collaboration. Analyzing Figure 3, it becomes apparent that the United States and the United Kingdom have made the most significant contributions to the literature in this particular field. Additionally, England, Wales, and Poland have demonstrated the highest levels of cooperation with other nations.

Table 2 showcases the leading 10 institutions in the field, ranked according to their publication output. Throughout the study period, the University of Reading took the lead with the highest number of papers (*n* = 18, 3.24%). Following closely behind were the University of San Francisco (*n* = 15, 2.70%), the University of Southampton (*n* = 14, 2.52%), and the University of Surrey (*n* = 14, 2.52%). Among these institutions, six hail from England, three from the United States, and one from Australia.

**TABLE 2:** Top 10 institutions by number of publications.

Rank	Institution	Publications,^a^ *n* (%)	Citations,^b^ *n* (%)	Total link strength	Average references, *n*
1	University of Reading (England)	18 (3.24)	365 (4.14)	7	20.27
2	University of San Francisco (United States)	15 (2.70)	143 (1.62)	40	9.53
3	University of Southampton (England)	14 (2.52)	337 (3.83)	29	24.07
4	University of Surrey (England)	14 (2.52)	250 (2.84)	18	17.86
5	University of Pittsburgh (United States)	11 (1.98)	123 (1.40)	37	11.18
6	The University of Manchester (England)	10 (1.80)	498 (5.65)	9	49.8
7	University of Washington (United States)	10 (1.80)	98 (1.11)	16	9.8
8	University of Aberdeen (England)	9 (1.62)	162 (1.84)	13	18
9	Flinders University (Australia)	8 (1.44)	80 (0.91)	27	10
10	Staffordshire University (England)	8 (1.44)	229 (2.60)	2	28.63

*^a^N* = 555.

*^b^N* = 8,808.

**FIGURE 4:**
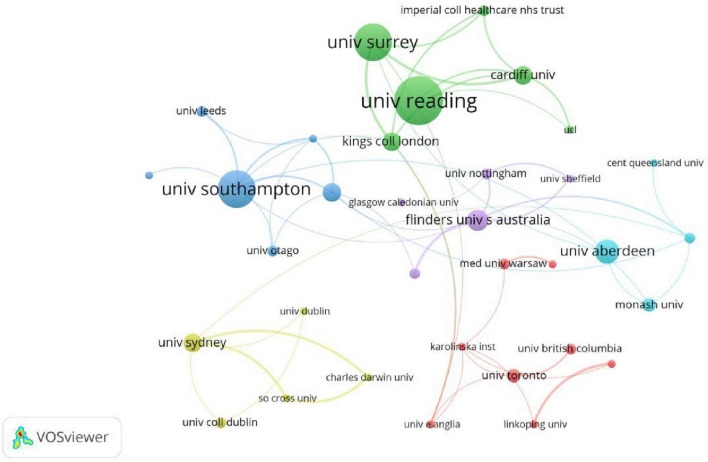
Institutional cooperative network (1989–2023).

The co-authorship relationships among institutions were analyzed, and a total of 97 institutions with strong associations were identified using VOSviewer. Figure 4 depicts the institutional cooperative network, which comprises six clusters, each represented by a different color. The size of each node corresponds to the number of publications, while the color of the circle indicates the clustering, and the thickness of the links represents the cooperation strength. For an institution to be included in the network, it had to have a minimum of four publications. The Green Cluster represents a collaborative network centered around the University of Reading, University of Surrey, and Cardiff University. Notably, the University of Surrey and King’s College London exhibit a high level of collaboration, as denoted by the thick line connecting them. The Blue Cluster represents a collaborative network centered around the University of Southampton. The Purple Cluster represents a collaborative network centered around Flinders University and the University of Nottingham. The Yellow Cluster represents a collaborative network centered around The University of Sydney. The Cyan Cluster represents a collaborative network centered around The University of Aberdeen and The University of Melbourne. These clusters predominantly consist of institutions from England and Australia. Lastly, the Red Cluster represents a collaborative network centered around the University of Toronto.

### Journals

The study analyzed a total of 555 articles published in 237 different journals. In Table 3, the top 10 journals for publication are presented, with information on total citation frequency and average citation frequency. The Journal of Advanced Nursing ranked first, with 39 papers accounting for 7.03% of all 555 papers. It also had the highest citation frequency of 735 times, representing 10.11% of the total citation frequency (*N* = 7,270). Following that, the Journal of Clinical Nursing (*n* = 32, 5.77%) was ranked second, followed by the Journal of the American Association of Nurse Practitioners (*n* = 24, 4.32%), and the Journal for Nurse Practitioners (*n* = 22, 3.96%). It is important to note that the rankings of journals based on published articles do not necessarily align with their corresponding citations and average citation counts, as indicated by the data in Table 3. Some journals with relatively few articles have higher citation counts and average citations. The average number of citations can serve as an indicator of the relative quality of papers published in different journals. Table 4 provides the top 10 most cited journals. Interestingly, some journals receive more citations despite having fewer publications. For example, the British Medical Journal was the most cited (*n* = 874, 12.02%), even though only four papers were published with an average of 218.5 citations each. This suggests that the papers published in this journal played a significant role in advancing research on nurses’ prescribing rights.

**TABLE 3:** Top 10 most productive journals.

Rank	Journal	Publications,^a^ *n* (%)	Citations,^b^ *n* (%)	Average references, *n*
1	Journal of Advanced Nursing	39 (16.46)	735 (10.11)	18.85
2	Journal of Clinical Nursing	32 (5.77)	573 (7.88)	17.91
3	Journal of the American Association of Nurses Practitioners	24 (4.32)	100 (1.38)	4.17
4	Journal for Nurses Practitioners	22 (3.96)	100 (1.38)	4.55
5	Journal of Psychiatric m and Mental Health Nursing	20 (3.60)	103 (1.42)	5.15
6	International Journal of Nursing Studies	12 (2.16)	280 (3.85)	23.33
7	Nurses Education Today	11 (1.98)	280 (3.85)	25.45
8	Journal of the American Academy of Nurses Practitioners	10 (1.80)	133 (1.83)	13.3
9	BMC Health Services Research	8 (1.44)	127 (1.75)	15.88
10	Archives of Psychiatric Nursing	7 (1.26)	74 (1.02)	10.57

*^a^N* = 555.

*^b^N* = 7,270.

**TABLE 4:** Top 10 journals by citations.

Rank	Journal	Citations,^a^ *n* (%)	Publications,^b^ *n* (%)	Average references, *n*
1	British Medical Journal	874 (12.02)	4 (1.69)	218.5
2	Journal of Advanced Nursing	735 (10.11)	39 (16.46)	18.85
3	Journal of Clinical Nursing	573 (7.88)	32 (13.50)	17.91
4	Journal of Psychiatric and Mental Health Nursing	280 (3.85)	20 (8.44)	14
5	Nurses Education Today	280 (3.85)	11 (4.64)	25.45
6	Journal of the American Academy of Nurses Practitioners	133 (1.83)	10 (4.22)	13.3
7	BMC Health Services Research	127 (1.75)	8 (3.38)	15.88
8	Family Practice	111 (1.53)	6 (2.53)	18.5
9	International Journal of Nursing Studies	103 (1.42)	20 (8.44)	5.15
10	Journal for Nurses Practitioners	100 (1.38)	22 (9.28)	4.55

^a^*N* = 7,270.

^b^*N* = 237.

### Author distribution

A total of 1,902 authors participated in the publication of 555 articles in this paper. Table 5 presents the top 10 authors based on their total citation frequency and average citation frequency. Courtenay ranked first with 31 papers, accounting for 5.59% of all 555 papers. Courtenay also had the highest citation frequency with 504 times, accounting for 3.02% of the total citation frequency (*N* = 16,679). Following Courtenay, Carey (*n* = 20, 3.60%), Stenner (*n* = 10, 1.80%), and Jones (*n* = 8, 1.44%) were also among the top authors. It is important to note that the ranking of authors based on published articles does not always align with their citation count or average citations. Some authors with fewer published articles have received high citation counts and average citations. The variation in average citation numbers may reflect the relative quality of papers published by these authors. The number of articles published by an author does not necessarily indicate the quality or popularity of their work. To further explore this, we examined the top 10 cited authors, as displayed in Table 6. It is evident that some authors have received more citations despite having fewer publications. For instance, Best, Dart, Grigg, Hare, and Ho published only 1 paper but received 279 citations, indicating the significant impact of their research on nurse prescription rights.

**TABLE 5:** Top 10 most effective authors.

Rank	Author	Publications,^a^ *n* (%)	Citations,^b^ *n* (%)	Total link strength	Average references, *n*
1	Courtenay	31 (5.59)	504 (3.02)	66	15.27
2	Carey	20 (3.60)	371 (2.24)	39	18.55
3	Stenner	10 (1.80)	194 (1.16)	43	19.4
4	Jones	8 (1.44)	70 (0.42)	28	8.75
5	Latter	8 (1.44)	137 (0.82)	17	17.13
6	Black	6 (1.08)	39 (0.23)	9	6.5
7	Blenkinsopp	6 (1.08)	68 (0.41)	1	11.33
8	Cashin	5 (0.90)	99 (0.59)	10	19.8
9	Gage	5 (0.90)	101 (0.61)	15	20.2
10	Buckley	5 (0.90)	27 (0.16)	13	5.4

*^a^N* = 555.

*^b^N* = 16,679.

**TABLE 6:** Top 10 authors by number of citations.

Rank	Author	Citations,^a^ *n* (%)	Publications,^b^ *n* (%)	Total link strength	Average references, *n*
1	Courtenay	504 (3.02)	31 (5.59)	1,111	15.27
2	Carey	371 (2.24)	20 (3.60)	813	18.55
3	Best	279 (1.67)	1 (0.18)	50	279
4	Dart	279 (1.67)	1 (0.18)	50	279
5	Grigg	279 (1.67)	1 (0.18)	50	279
6	Hare	279 (1.67)	1 (0.18)	50	279
7	Ho	279 (1.67)	1 (0.18)	50	279
8	Jelinek	248 (1.49)	1 (0.18)	44	248
9	Mcneil	248 (1.49)	1 (0.18)	44	248
10	Newman	248 (1.49)	1 (0.18)	44	248

*^a^N* = 16,679.

^b^*N* = 555.

We utilized VOSviewer to analyze the co-authors, resulting in a network comprising 1,902 authors, each having published more than one article (Figure 4). In the figure, color denotes clustering, node size indicates the number of published documents, and link thickness represents the strength of cooperation. A total of six author cooperation networks have emerged. Among them, the purple cluster stands out as one of the most significant, featuring Courtenay (31 papers, total link strength 66), Carey (20 papers, total link strength 39), and Stenner (10 papers, total link strength 43). The team’s research primarily revolves around analyzing barriers and facilitators in the implementation of nurses’ prescription rights in diabetic patients, as well as discussing its application effectiveness.

### Research category

Table 7 illustrates the top 10 categories of prescriptions issued by nurses. The data for these categories were obtained from the WoS database system through search results. Among the various study categories, the three most prominent ones concerning nurses’ prescribing rights were Nursing (317/555, 57.12%), Health Care Sciences Services (82/555, 14.78%), and Medicine General Internal (59/555, 10.63%).

**TABLE 7:** Top 10 research categories.

Rank	WoS^a^ categories	Publications, *n* (%)
1	Nursing	317 (57.12)
2	Health Care Sciences Services	82 (14.78)
3	Medicine General Internal	59 (10.63)
4	Psychiatry	49 (8.83)
5	Public Environmental Occupational Health	44 (7.93)
6	Health Policy Services	32 (5.77)
7	Pharmacology Pharmacy	26 (4.69)
8	Substance Abuse	21 (3.78)
9	Primary Health Care	18 (3.24)
10	Pediatrics	14 (2.52)

### Keywords

Keywords play a critical role in analyzing the knowledge structure of an academic field using bibliometrics, as they assist in identifying potential research hotspots (15). Therefore, the focus of this study is to determine the covered topics by analyzing these keywords. In Table 8, the top 25 keywords are presented based on their occurrence. Among them, the keyword “Nursing practitioners” is the most frequently mentioned, appearing 90 times and having a total link strength of 415. Additionally, “Nursing prescribing,” “Nursing,” “Prescribing,” “Opioids,” “Primary care,” and “Education” are also highly ranked keywords in terms of their frequency.

**TABLE 8:** Top 25 keywords in the study of nurses’ prescribing rights from 1989 to 2023.

Rank	Keyword	Count	Total link strength
1	Nursing practitioners	90	415
2	Nursing prescribing	76	284
3	Nursing	73	340
4	Prescribing	72	306
5	Opioids	38	152
6	Primary care	38	181
7	Education	29	126
8	Buprenorphine	24	113
9	Survey	23	103
10	Medication management	22	102
11	Advanced practice registered nursing	20	88
12	Non-medical prescribing	20	85
13	Policy	19	86
14	Scope of practice	19	79
15	Mental health	17	69
16	Antibiotics	16	66
17	Opioids use disorder	15	63
18	Pain management	15	69
19	Prescriptive authority	15	60
20	Advanced practice	14	56
21	Physician assistants	13	72
22	Pharmacology	12	42
23	Chronic pain management	11	48
24	Diabetes	11	46
25	Barriers	10	58

**FIGURE 5:**
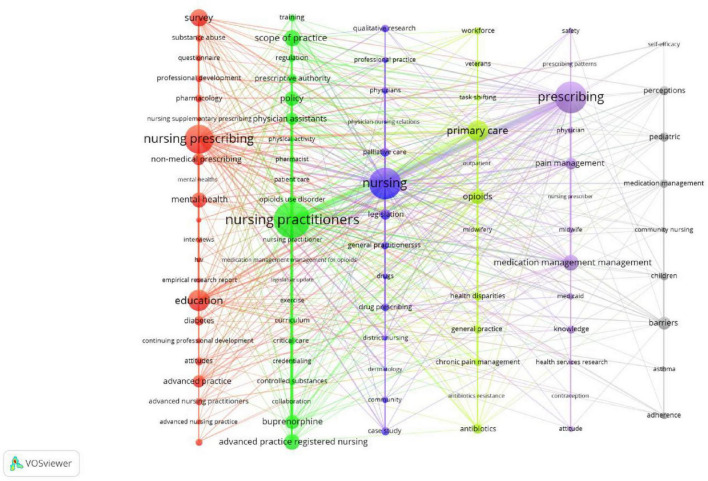
Keyword co-occurrence network (1989-2023).

We conducted an analysis of keywords that appeared more than 4 times in the titles and abstracts of 555 papers. Utilizing a clustering algorithm similar to modularity-based clustering, we identified the top 93 keywords and visually represented them. In Figure 5, the visualization showcases different clusters using distinct colors. Nodes of the same color belong to the same cluster, and the size of the nodes indicates the number of corresponding keywords. The thickness of the links between nodes represents the co-occurrence strength, while the thickness of the lines between nodes indicates the collinearity strength between keywords. We categorized the keywords into six color-coded categories: red, green, blue, yellow, purple, and gray. This categorization accentuates the prominent research hotspots and cutting-edge fields within these topics.

Cluster 1 focuses on the education and training of nurses regarding prescribing rights. The primary keywords include “Nursing prescribing,” “Education,” “Survey,” “Mental health,” and “Non-medical prescribing.” This cluster primarily explores the development of educational programs for nurses to acquire prescription rights. Cluster 2 centers around Nursing practitioners. The main keywords within this cluster are “Nursing practitioners,” “Scope of practice,” “Policy,” “Advanced practice registered nursing,” and “Buprenorphine.” The main focus of this cluster is the examination of policies related to nurses’ prescribing rights. Cluster 3 is associated with Palliative care. The main keywords in this cluster include “Nursing,” “Palliative care,” “Legislation,” “Case study,” and “Drug prescribing.” This cluster primarily investigates the application of nurse prescribing rights in the context of Palliative care. Cluster 4 revolves around Primary care. The main keywords within this cluster are “Primary care,” “Opioids,” “Antibiotics,” “Chronic pain management,” and “General practice.” This cluster primarily explores the application of nurse prescribing rights in the domain of Primary care. Cluster 5 focuses on Pain management. The main keywords in this cluster are “Prescribing,” “Pain management,” “Medication management,” “Knowledge,” and “Attitudes.” This cluster primarily examines the utilization of nurse prescribing rights in the field of Pain management. Cluster 6 is associated with Barriers. The main keywords within this cluster include “Barriers,” “Pediatric,” “Perceptions,” and “Children.” This cluster primarily investigates the barriers and facilitators of implementing nurse prescribing rights in pediatric settings and discusses the impact of its application. These six clusters constitute the main areas of study regarding nurse prescribing rights.

**FIGURE 6:**
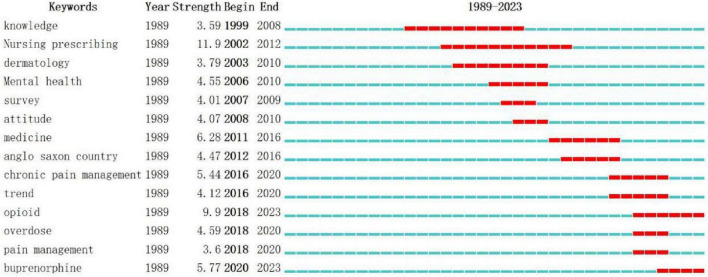
Top 14 keywords with strong citation bursts between 1989 and 2023.

Keywords that experience sudden and widespread citation or are cited in large numbers within a short period of time are known as burst keywords (11). These burst keywords are identified using CiteSpace’s default Kleinberg algorithm and serve as important indicators of cutting-edge research hotspots, with sudden keywords indicating emerging trends. In Figure 6, the top 14 burst keywords in this field from 1989 to 2023 are illustrated. The thick red line represents the time period during which the keyword experiences a burst. Among these burst keywords, “Nursing prescribing” (11.9) exhibits the highest intensity, followed by “Opioids” (9.9). Notably, “Nursing prescribing” stands out with the longest outbreak time, spanning 10 years. Recent emergent keywords such as “Opioid,” “Overdose,” “Pain management,” and “Buprenorphine” also reflect the current research trends in this field and have the potential to become future research hotspots.

### Articles with high citations

To some extent, the citation ranking of papers can offer valuable insights into the prominent research areas within the academic domain (16). In addition to that, by examining the co-occurrence of keywords and emerging terms, we can gain further understanding of highly cited articles. In Table 9, we have compiled a list of the top 10 articles based on their citation count. Our analysis reveals that these 10 articles predominantly cover 4 significant research topics: Buprenorphine, Emergency departments, Dermatology, and Palliative care.

**TABLE 9:** Top 10 cited articles.

Rank	Title (author)	Year	Citations	Journal
1	Nurse prescribing of medicines in Western European and Anglo-Saxon countries: a systematic review of the literature (17)	2011	108	BMC Health Services Research
2	The effects of nurse prescribing: a systematic review (18)	2014	91	International Journal of Nursing Studies
3	Effectiveness of nurse prescribing: a review of the literature (19)	2004	73	Journal of Clinical Nursing
4	Nurse practitioner independence, health care utilization, and health outcomes (13)	2018	71	Journal of Health Economics
5	In rural areas, buprenorphine waiver adoption since 2017 driven by nurse practitioners and physician assistants (20)	2019	65	Rural Health
6	Overcoming barriers to prescribing buprenorphine for the treatment of opioid use disorder: recommendations from rural physicians (21)	2019	64	The Journal of Rural Health
7	Uganda: delivering analgesia in rural Africa: opioid availability and nurse prescribing (22)	2007	63	Journal of Pain and Symptom Management
8	Impact of nurse prescribing: a qualitative study (12)	2007	62	Nursing and Health Sciences
9	Patients’ views of nurse prescribing: effects on care, concordance and medicine taking (23)	2011	61	British Journal of Dermatology
10	Nurse prescribing of medicines in Western European and Anglo-Saxon countries: a survey on forces, conditions and jurisdictional control (6)	2012	60	International Journal of Nursing Studies

## Discussion

Our research shows that over the past 34 years, the number of studies on nurses’ prescription rights has steadily increased, with a notable surge in the last decade. This trend underscores the growing recognition of the role of nurses in prescribing within the global healthcare system. However, as an interdisciplinary issue encompassing both policy and practice, the research and development of nurses’ prescription rights has progressed at a relatively slow pace, leaving substantial room for further advancement.

From a national and regional perspective, research on nurses’ prescription rights is concentrated in a few high-income countries. The United States has the highest number of published papers in this area, while the United Kingdom leads in terms of total citation frequency and the strength of international collaborations. This disparity may be linked to the earlier introduction of nurse prescription legislation and the establishment of a supportive national policy environment. In contrast, research on nurses’ prescription rights is relatively scarce in low- and middle-income countries, where international collaborative networks are also weaker. This imbalance is likely influenced by various factors, including the level of national economic development, the intensity of health policy promotion, and disparities in education and training systems.

Further analysis indicates that in high-income countries, the expansion of nurses’ prescription rights is not only evident in the increasing number of studies, but also in actual policy changes and improvements in healthcare service efficiency. For example, the enactment and revision of several policies in the United States, such as the Addiction Recovery Act of 2017, which allows practicing nurses to prescribe controlled substances, occurred before and after the significant growth in research on nurses’ prescription rights (14). This suggests that policy changes play a crucial role in driving research interest.

In this context, the practical and political implications of expanding nurses’ prescription authority must be urgently addressed in low- and middle-income countries. These countries typically face challenges such as limited medical resources, a shortage of doctors, and weak primary healthcare systems. Granting nurses appropriate prescription authority could significantly alleviate the pressure on primary healthcare services.

Although bibliometric data show that low- and middle-income countries remain on the periphery in terms of publication volume and collaborative networks, this does not imply that nurses’ prescription rights are unimportant in these regions. On the contrary, the weak foundation of related research underscores the need to advance the field through policy initiatives and to promote international academic and practical cooperation for knowledge sharing and experience transfer. Future research should focus on areas such as the implementation pathways of nurses’ prescription rights in resource-limited settings, the development of education and training systems, cost–benefit analyses, and patient health outcomes, to address the existing research gaps.

In terms of institutional contributions, the University of Reading ranks first, while the University of Manchester excels in terms of total citations in the study of nurses’ prescribing rights. The Journal of Advanced Nursing is the prominent publication with the highest number of articles on this topic, while the British Medical Journal leads in terms of total article citations. Courtenay et al. (23) have made significant contributions in terms of both the number of articles and citation frequency. Subject analysis highlights Nursing, Health Care Sciences Services, and Medicine General Internal as the three most important research areas pertaining to nurse prescribing rights.

The keywords serve as highly condensed and summarized focal points of research within a specific field (15). By summarizing frequently occurring keywords and examining the clusters they form, along with analyzing the content of the top 10 highly cited literature sources, we can conclude that the primary research areas regarding nurses’ prescribing authority revolve around Nursing practitioners, Primary care, and Education. Emergent words refer to rapidly emerging keywords that surpass high-frequency ones in predicting research trends at the forefront (11). Through an analysis of emergent words, it has been determined that the current forefront of research on nurses’ prescribing rights predominantly focuses on Opioids (2018–2023) and Buprenorphine (2020–2023).

### Nursing practitioners

The granting of prescribing rights primarily applies to senior practice nurses, especially Nursing Practitioners. The emergence of Nursing Practitioners has significantly contributed to the expansion of nurses’ prescribing authority. Research conducted by Gagan et al. (24) and Carey et al. (25) demonstrates that the diagnostic and treatment services provided by nursing practitioners can greatly enhance patients’ understanding of chronic diseases, improve their ability to manage their health, enhance adherence to guidelines and treatment, improve their quality of life, and reduce the incidence of acute attacks and readmissions for chronic diseases. Additionally, it increases patients’ satisfaction with their diagnosis and treatment. Nursing practitioners also assume emergency tasks in large hospitals. Surveys conducted by Plath et al. (26), Griffin and McDevitt (27), and Thrasher and Purc-Stephenson (28) in Australia, Ireland, and Canada reveal that nursing practitioners reduce waiting and treatment times for emergency patients, resulting in increased patient satisfaction. Furthermore, there is no significant difference in treatment outcomes between nursing practitioners and doctors. Notably, 99.1% of patients expressed their willingness to choose nursing practitioners again. Sandhu et al. (29) investigated the disparities in doctor-patient communication skills and patient satisfaction between emergency nursing practitioners and doctors. The findings indicate that patients treated by nursing practitioners exhibit higher levels of satisfaction compared to those treated by doctors. Additionally, nursing practitioners excel in selecting appropriate communication methods and treatment plans based on patients’ education and medical conditions. However, some studies (30) have identified challenges in the practice of nursing practitioners. Some nursing practitioners perceive difficulties in treating patients with multiple chronic conditions, which may be attributed to their limited professional knowledge and self-confidence.

### Primary care

Nurse prescribing rights in the United Kingdom were legalized in 1992, originating from community healthcare. These rights are primarily granted to senior practice nurses, particularly nursing practitioners. Throughout the development process, as well as currently, community health service centers have been the preferred practice settings for nursing practitioners to provide medical services. Recent data from 2018 in the United States indicates that nursing practitioners are more likely to practice in communities, rural areas, and remote locations, with a higher willingness to work in community health service centers compared to doctors. Research (31, 32) reveals that 87% of nursing practitioner graduates choose community settings for their practice, while only 8% of medical students opt for residency training in general practice in the same year. A systematic review of 18 studies conducted in 7 countries, including the United Kingdom, the United States, and the Netherlands (33), demonstrated that compared to doctor-led treatment, nursing practitioners had slightly better outcomes in terms of case fatality rate, physiological indicators, and patient satisfaction. There were no statistically significant differences in prescriptions, medical errors, referrals, and the number of hospitalized patients between nursing practitioners and doctors. Additionally, nursing practitioners spent more time on average treating patients and had a higher rate of return visits compared to doctors.

### Education

Different countries have their own education and training systems for nurses. In countries like the United States, Australia, and Canada, nursing programs at the master’s degree level include relevant training. For example, in Australia, students are required to complete 5,000 h of advanced coursework and prescription training, with core courses covering pharmacology, physical examination, laboratory examination, and radiation technology (34). On the other hand, Finland and Spain incorporate nurse prescribing knowledge into their undergraduate degree programs (35, 36). In contrast, countries like the United Kingdom, Ireland, and Sweden provide separate training for nurses to acquire prescription rights. In Ireland, registered nurses and midwives must complete a rigorous 6-month theoretical and practical course and pass an assessment that covers various aspects including pharmacology, prescription writing, and pharmacodynamics (37). However, due to the broad scope of prescribing training in nursing practice, several countries focus on general knowledge modules such as pharmacokinetics, pharmacodynamics, advanced health assessment, and prescribing principles. Nevertheless, studies have shown that nurses have a demand for specialty-specific prescription courses (38). Instructors of prescription courses also believe that courses should be tailored to different specialties to better align course content with clinical practice, enhance nurses’ proficiency in prescription knowledge and skills in their specific fields, and facilitate their transition from learners to practitioners.

### Buprenorphine and opioid

The United States continues to grapple with an ongoing opioid overdose crisis, as many individuals still lack access to effective treatment (39). Buprenorphine, a widely used and effective treatment for opioid use disorder (OUD), is readily available in medical offices as a partial opioid agonist (40). However, the utilization of buprenorphine therapy for OUD patients remains limited, partly due to the scarcity of buprenorphine prescriptions (41, 42). In 2017, the United States implemented the Comprehensive Addiction and Recovery Act, granting nurse practitioners the authority to prescribe buprenorphine (14). Since the enactment of this program, nurse practitioners have played an increasingly vital role in providing effective treatment for OUD in communities. Between 2016 and 2019, over 12,000 nurse practitioners and physician assistants gained the privilege to prescribe buprenorphine, resulting in a remarkable 111% increase per 100,000 people in rural areas. Nurse practitioners and physician assistants accounted for more than half of the newly issued prescriptions (20). Nonetheless, nurses, like doctors, encounter various factors that either promote or hinder their ability to prescribe buprenorphine. Promotion factors include possessing prescribing skills, having mentors, and feeling rewarded, while barriers encompass regulation, inadequate education, and stigma (43–46). A study by Chandra revealed that the main obstacles faced by nurses in prescribing buprenorphine were stigma and the lack of reimbursement for expenses related to OUD (47).

This study systematically reviews and analyzes research on nurses’ prescription rights using bibliometric methods, making several important contributions. First, the findings hold significant value for planning nursing education. By revealing the developmental trajectory and research hotspots of nurses’ prescription rights both domestically and internationally, this study provides valuable data to support curriculum design and talent cultivation. Such insights can assist educational administrators and instructors in more effectively training nursing professionals with advanced practical competencies.

Secondly, this study also provides valuable guidance for the formulation of health policies. As nurses’ prescribing authority is increasingly being promoted worldwide, policymakers require a comprehensive understanding of the current status and emerging trends in academic discussions surrounding this issue. By referencing scientific evidence revealed through this bibliometric analysis, policymakers can make more informed decisions when formulating relevant laws and regulations. This, in turn, will better support the development of the nursing profession and contribute to the continuous improvement of healthcare systems.

Finally, through a statistical analysis of the existing literature, this study identifies the shortcomings and gaps in current scientific research on nurses’ prescription authority. For instance, research from low- and middle-income countries remains limited, and the assessment of policy implementation outcomes requires further investigation. These findings highlight clear directions and areas for improvement for future academic research and practical initiatives.

In conclusion, this study holds practical significance and reference value in areas such as nursing education planning, health policy development, and the identification of gaps in scientific research. Furthermore, it provides a theoretical foundation and empirical data to support future research and practice regarding nurses’ prescribing rights.

The study has several limitations that should be acknowledged. Firstly, our data selection was focused on the core set of WoS, omitting other databases like Scopus. This choice may have excluded valuable research from our analysis, especially emerging research. Additionally, our study primarily examined journals, while other forms of scientific knowledge dissemination, such as books, working papers, and reports, received less attention. This narrow focus may limit the comprehensiveness of our findings. Secondly, there are subjective factors involved in our sample selection. For instance, we only analyzed English-language publications, which introduces a potential linguistic bias. To gain a more comprehensive view, it would be beneficial to compare articles published in different languages or countries. Furthermore, subjectivity may have influenced our search strategy and filtering, as it is challenging to completely eliminate bias in bibliometric research. Lastly, our research primarily focused on bibliometric analysis, aiming to analyze the knowledge structure in the field of nurses’ prescribing rights. However, this study utilized only bibliometric methods to visualize the objective characteristics of nurses’ prescribing rights. The study did not explore the substantive content related to nurses’ prescribing rights, indicating the need for a more comprehensive literature review in future research. Overall, these limitations should be considered when interpreting our findings. Future studies should address these issues to enhance the validity and scope of the research.

## Conclusion

Our study reveals a gradual increase in the number of publications on nurse prescribing rights over the past 34 years. However, the field is progressing at a relatively slow pace, leaving ample room for further development. Moreover, research advancement is uneven across different countries and regions, with limited collaboration among those with less developed research fields. Notably, the United States has emerged as the leading contributor in terms of published papers on nurses’ prescribing rights. Meanwhile, the United Kingdom takes the lead in both the total number of literature citations and the strength of partnerships. In terms of subject analysis, Nursing, Health Care Sciences Services, and Medicine General Internal stand out as the three key research areas pertaining to nurses’ prescribing rights. The research primarily focuses on Nursing practitioners, Primary care, and Education. Moving forward, the research frontiers in nurse prescribing rights are expected to revolve around Opioids (2018–2023) and Buprenorphine (2020–2023).

## Data Availability

The original contributions presented in this study are included in this article/supplementary material, further inquiries can be directed to the corresponding authors.
